# Stephanie Beaupark Sees Chemistry Through an Indigenous Lens

**DOI:** 10.1021/acscentsci.4c02023

**Published:** 2024-12-17

**Authors:** Jonathan Feakins

Describing the benefits of combining laboratory-based techniques and Indigenous knowledges, Stephanie Beaupark harks back to her experience as a weaver. Much like how she once wove *Lomandra* grass together to create ropes as a collaborating artist at the University of Melbourne, Beaupark hopes that mixing the two distinct traditions of knowledge acquisition can create something stronger.

Her PhD research at the University of Wollongong aims to uncover how the colors of Australian indigenous dyes vary as the seasons change. Because she’s a chemist by training, part of her approach involves modern molecular analysis—a technique that exemplifies what is commonly known as Western science, a framework that tries to rigorously categorize the natural world through experimentation. But she is also relying on traditional Indigenous knowledges.

In her case, that means relying on connections that Aboriginal and Torres Strait Islander peoples have built with the continent over at least 65,000 years. Beaupark’s investigations into eucalyptus dyes may provide insight into the chemical underpinnings of eucalyptus’s myriad uses not only as a dye but as timber, medicine, smoke for signaling or spiritual ceremonies, and more.

A Ngugi woman, Beaupark makes deliberate efforts to collaborate and partner with Aboriginal communities in Australia. In part because of this approach, she received the Australian Academy of Science Aboriginal and Torres Strait Islander Scientist Award last year, which recognizes emerging Aboriginal and Torres Strait Islander scientists, to fund the budding project.

Beaupark’s work illustrates how environmental chemistry can benefit from researchers’ long-term interactions with the land and with Indigenous peoples just as much as it benefits from robust data collected over the past century. And yet, knowledges from Indigenous communities—including their Elders—also hold a deeper kind of value that cannot be so easily quantified.

## How to gather knowledge

Beaupark’s approach is exemplified by climate research she published last year. During an internship at the University of Wollongong Centre for Atmospheric Chemistry, she studied how the knowledge of seasons of Darug people, an Aboriginal group native to what is now western Sydney, might relate to annual cycles in air quality.

While atmospheric science assumes four annual seasons, Beaupark argued that this model is a European import awkwardly applied to the Australian climate. Taking into account Darug people’s Traditional Knowledge and doing her own statistical analysis of temperature, wind speed, and wet versus dry weather, Beaupark ultimately categorized annual weather cycles in the Sydney Basin into six seasons of varying lengths.

In her eventual paper, she coined the term IKALC-seasons (Indigenous knowledge applied to local climatology), named in consultation with Indigenous coauthors. For example, Beaupark’s team found that the highest annual levels of carbon monoxide, nitrogen oxides, and airborne particulate matter coincided with a “cold and still” IKALC-season that stretches from early May to late July.

As Beaupark starts the eucalyptus project, uplifting Indigenous frameworks of knowledge is central to her approach. She deliberately opts to pursue her PhD part-time on top of working full-time as a lecturer, a pace that feels right for what she’s doing. “The timeline is a lot slower than what is often required in Western science. It’s about maintaining relationships with people. It’s not just for the project,” Beaupark says. “Once you make a connection with the community, you’re in it for life. That’s the cultural way of doing things.”

## A fading planet

Beaupark remembers feeling inspired and concerned when she first came across a map of Australia created by Lao Australian artist Samorn Sanixay. The bulk of Australia’s northern latitudes teemed with beige and tan hues, but the country’s southern, more temperate zones quickly gave way to rich oranges and reds until, at the very southern tip of Tasmania, the map became almost mahogany.

The map, made of dozens of bundles of dyed wool, was the product of Sanixay’s yearlong expedition to create an atlas of local eucalyptus pigments. For Sanixay’s project, dubbed Eucalypt Dye Catalogue, she gathered dye samples of more than 300 eucalyptus species as part of a yearlong fellowship with Eucalypt Australia. As she did so, she spoke with local Aboriginal peoples whose diversity—more than 500 distinct groups—rivals that of eucalyptus itself.

**Figure:**
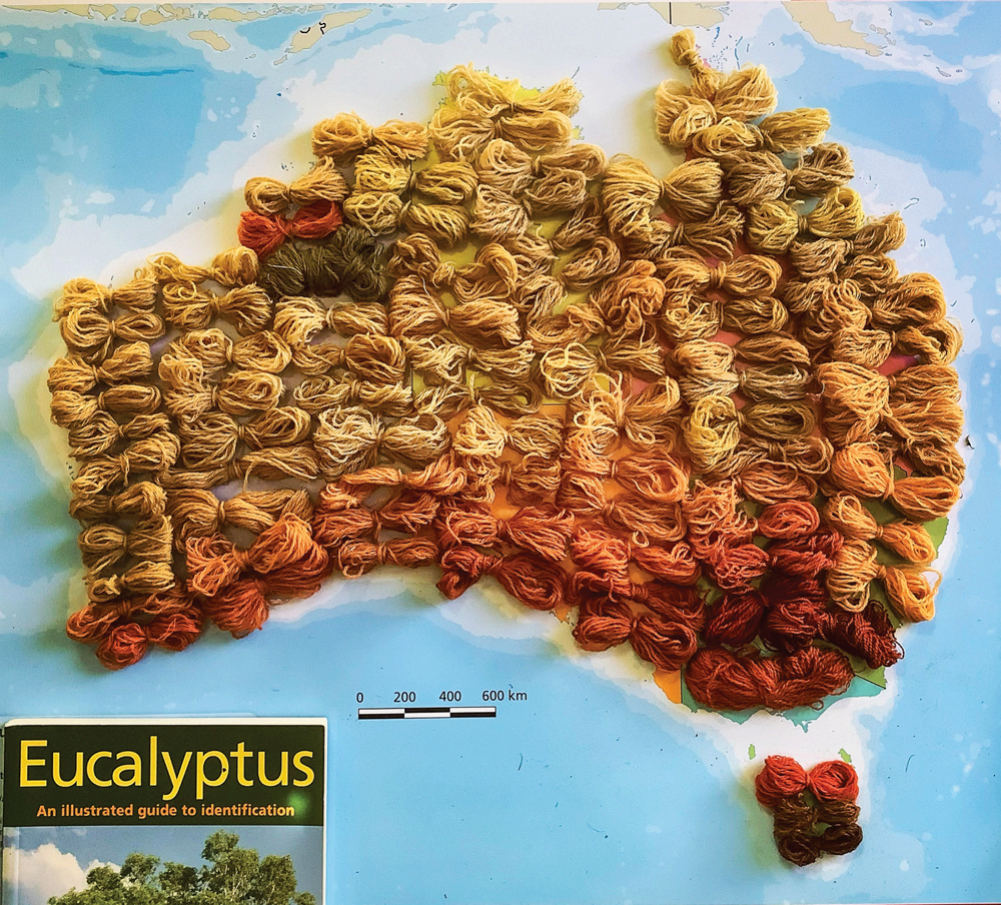
For her Eucalypt Dye Catalogue, Samorn Sanixay created her very own map of Australia made of eucalyptus dye. Credit: Samorn Sanixay.

The spectrum of dyes demonstrates a clear connection between latitude and color. But more pressing, it shows how eucalyptus dyes may adapt to the rising temperatures and recurrent droughts that climate change causes; the dyes may adopt the neutral hues found in arid inner regions instead of the rich, chocolate reds that pepper the southern coast.

After seeing Sanixay’s work online, Beaupark asked her to participate in a yarning session for Beaupark's PhD research and to learn about her travels.

“That map could give us a glimpse into the future, if we continue the way we currently live in this world: damaging Country, not caring for Country as if it was our family the way Indigenous people have always done; living on the landscape and not alongside it,” Beaupark says. “It’s not about a hierarchical structure of putting people first,” in which human beings exert dominion over the environment and consider its needs as secondary to their own. When entering into a mutually respectful relationship with Country, tending to and observing the environment are paramount.

(Aboriginal and Torres Strait Islander peoples use the term *Country* to refer to the lands and waters to which they’re connected. The word also carries complex connotations related to spiritual and cultural practice, language, law, and family.)

Beaupark is now applying her chemistry lab know-how and her experience gathering Indigenous knowledges to understand how pigments change in eucalyptus.

## A pigment’s purpose

To make eucalyptus dyes in the lab, Beaupark follows the same process she does when making art, boiling the tree’s leaves and smaller connecting branches. “What’s extracted in the dyes is the secondary metabolites,” Beaupark says. These colorful metabolites, such as eucalyptus’s polyphenols, carotenoids, and terpenoids, give the tree its color and impart adaptations to environmental factors like ultraviolet radiation and bacterial infections. “They’re all the things that are helping the tree survive. Those things will adapt as the landscape changes. And that’ll change the colors,” Beaupark says.

**Figure:**
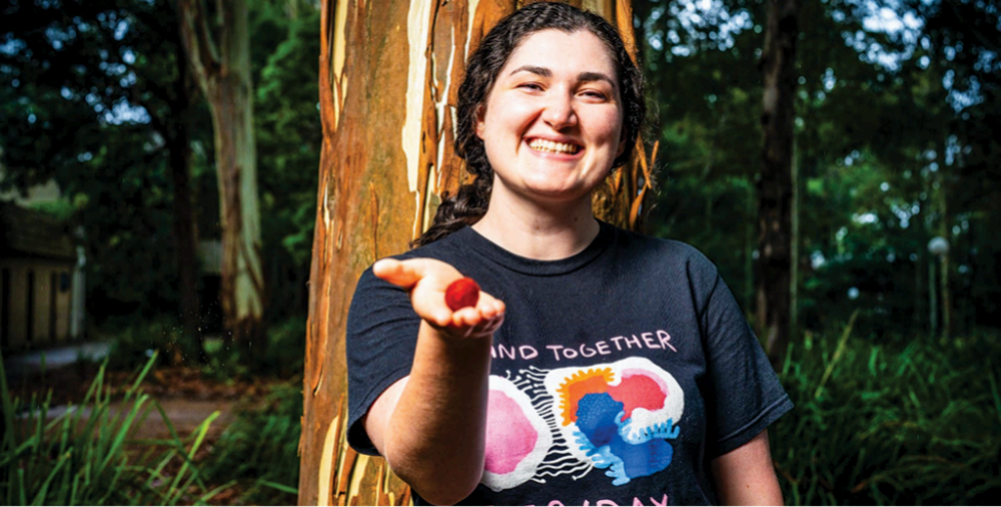
Stephanie Beaupark is working to analyze the chemistry of natural dyes made from eucalyptus plants. Credit: Paul Jones/University of Wollongong.

Take xanthophylls, a major family of plant pigments that contributes notes of yellow orange to eucalyptus leaves. These compounds’ primary role is modulating incoming energy from sunlight. According to a study done at Western Sydney University using simulated heat waves, eucalyptus can accelerate the de-epoxidation of its xanthophylls to help protect against photooxidative damage. This small molecular tweak shifts the pigments’ chemistry and thus may also affect the color of dyes derived from the trees.

Matthew Koski, a plant biologist at Clemson University, has studied climatic variations in plant pigments, particularly in response to ultraviolet radiation. Combing through plant archives across multiple continents, including Australia, Koski has uncovered an invisible battle. Since humans began shaping the planet’s atmospheric chemistry, plants have raced to upregulate and downregulate UV-absorbing pigments, largely in response to the degradation of Earth’s ozone layer.

One of the primary goals of these pigments is to protect plants’ evolutionarily precious pollen. “Most researchers in floral evolution and ecology focus on how pigments give rise to flashy colors that attract pollinators,” Koski says. Throughout the plant, these pigments are extremely important for providing protection from abiotic stress too: photostress, extreme temperatures, drought. “You can be as brightly pigmented and attract as many pollinators as you want—but if the gametes in the flower are inviable, that’s not going to lead to reproductive success,” Koski says.

Koski’s data set has limits, however. For one thing, it consists only of pigments stored in botanical archives, whose samples are typically less than a century old. So while these samples could shed light on how plants changed before and after some ozone-damaging chemicals were phased out by the 1987 Montreal Protocol on Substances That Deplete the Ozone Layer, it can’t illuminate trends that occurred before the archives’ inception.

Koski also cautions that it rarely behooves ecologists to seek out one-to-one relationships between the climate and plant traits. He does suspect, however, that areas experiencing rising temperatures—Australia included—will see a trend of plants losing some pigmentation in an attempt to absorb less solar radiation.

## Weaving fields together

What sets Beaupark’s lab apart is that it does not intend to study the dyes’ components in isolation. “Traditionally in natural products chemistry, the goal is to isolate and characterize new compounds,” Beaupark says. “But in my work, I have chosen to prioritize the knowledge of the tree holistically.” Beaupark obtains her samples from one specific *Eucalyptus cinerea* tree with which she says she feels a personal connection.

After making the dyes, her team analyzes the mixture with various techniques, such as liquid chromatography/mass spectrometry and colorimetric assays, which give “a bird’s-eye view of the components to understand any trends of the mixture across Indigenous seasons,” Beaupark says. She aims to measure how total phenolic, flavonoid, and proanthocyanidin contents of the dyes fluctuate throughout the year. Currently, she is still within the first year of her project and intends to save details of her discoveries until publication.

Indigenous knowledges don’t encode data the way a spreadsheet or ice core does, Beaupark says. “Looking at a specific tree, the knowledge would be about the relationships that tree has with the environment: all of the plants and animals that live around it, and the family of trees it is within. It’s more about wider context than this hyperspecific knowledge,” Beaupark says. “That’s a difference between Indigenous knowledge and Western science.”

Non-Indigenous researchers are also reckoning with their responsibilities when interfacing with Indigenous cultural heritage. Loïc Bertrand—a researcher in cultural heritage chemistry at the University of Paris-Saclay—first considered the responsibilities of working ethically with Indigenous communities while studying rock art in South Africa.

The rock art project required more consideration with local groups—not just acknowledging contemporary communities but working with them to understand how and why the art was made or placed where it was. In contrast, “the collections that we are studying in Europe, in certain respects, are dead,” Bertrand says.

A more recent project he worked on used high-resolution X-ray Raman spectroscopy to gather atomic-level information on historical Australian plant exudates, including those from eucalyptus. Much like the rock art in South Africa, the Australian exudates studied by Bertrand and Rachel Popelka-Filcoff, an archeological scientist then at Flinders University and now at the University of Melbourne, remain connected to a contemporary Aboriginal community.

As such, the researchers had to tailor their approaches appropriately—for example, by focusing strictly on the exudates’ historical aspects. Studying the plants’ modern uses or applications, by contrast, would likely have required exponentially more detailed and nuanced discussions with local communities, which the researchers could not have responsibly completed within the project’s timeline.

In other instances, researchers might conduct the destructive analysis on a modern model of the historical sample—for example, analyzing a sample of *Eucalyptus largiflorens* collected in the 21st century as opposed to one in an irreplaceable 19th-century archive. In an extension of her team’s initial research, Popelka-Filcoff is now working directly with Traditional Owners to source samples: “that is, going onto Country, talking with people, asking about what it’s called in a local language and how it is used.”

“We will present the analytical, synchrotron-based work, and then people may share Indigenous knowledge with us,” Popelka-Filcoff says. “It then becomes this two-way conversation, where there’s a lot of learning on both sides.”

Beaupark says that, with proper care and forethought, Western science can interface with Indigenous knowledges in a way that benefits all parties instead of following a one-sided, extractive model. She stresses “prioritizing Indigenous leadership and creating protocols and a shared understanding and open communication [about] how the knowledge is going to be used and how the project is going to be done.”

## The knowledge is always there

Beaupark is working with two Aboriginal groups to which she has strong ties: the Dharawal, who hail from the region where she now lives and works, south of Sydney, and people within her own Quandamooka Country, whose ancestral lands consist of the bay and islands just outside Brisbane. Beaupark ultimately hopes that her PhD might help plant the seed for a “community of research.”

For example, she pays artists with relevant cultural backgrounds to join her in making art and yarn “as a means of generating new knowledge.” And she says she recently hired two early career Indigenous research assistants to work alongside her in her lab.

“No matter where they go,” Beaupark says, “the fact that they’ve had this experience in the lab that is also grounded in culture—working with the plants, thinking about the dye mixture and about wider relationships—I think that’s really empowering, as an Indigenous scientist.”

As for her ongoing research into how Australian seasons affect the dyes derived from her tree, Beaupark looks forward to the “heaps of data analysis” that await her. She is “fairly confident,” when all is said and done, that “the science will catch up to the knowledge.”

She points to examples like modern research on mycorrhizal networks, which echoes teachings that predate laboratory science about how trees communicate and even respond as a family unit to damage against an individual tree. Even in a world where colonialism wreaked havoc on Aboriginal and Torres Strait Islander communities and their oral histories, Beaupark asserts that Indigenous knowledges are more dynamic and enduring than practitioners of Western science might think.

“It’s not that that knowledge is lost forever. The knowledge is held within the landscape,” Beaupark says. “It’s a long-term learning, alongside Country. It’s the same as the fundamental laws of matter. It can’t be created or destroyed: it’s always there.”

## Jonathan Feakins is a freelance contributor to

Chemical & Engineering News, *the independent news outlet of the American Chemical Society.*

